# Using a Three-Dimensional Reduction Method in the High-Efficiency Grain Selector and Corresponding Grain Selection Mechanism

**DOI:** 10.3390/ma12111781

**Published:** 2019-06-01

**Authors:** Xintao Zhu, Fu Wang, Shuaipeng Zhang, Tobias Wittenzellner, Jessica Frieß, Dexin Ma, Andreas Bührig-Polaczek

**Affiliations:** 1 Foundry Institute, RWTH Aachen University, 52072 Aachen, Germany; f.wang@gi.rwth-aachen.de (F.W.); zspdeutsch66106@gmail.com (S.Z.); t.wittenzellner@gi.rwth-aachen.de (T.W.); j.friess@gi.rwth-aachen.de (J.F.); d.ma@gi.rwth-aachen.de (D.M.); sekretariat@gi.rwth-aachen.de (A.B.-P.); 2 State Key Laboratory for Manufacturing System Engineering, School of Mechanical Engineering, Xi’an Jiaotong University, Xi’an 710049, China

**Keywords:** grain selector, reduction the dimensionality, 2D grain selector, Ni-based single crystal superalloys

## Abstract

In the development of a high-efficiency grain selector, the spiral selectors are widely used in Ni-based single crystal (SX) superalloys casting to produce single crystal turbine blades. For the complex three-dimensional structure of the spiral, a 2D grain selector was designed to investigate in this paper. As a result, the parameters of two-dimensional grain selection bond and the corresponding grain selection mechanism were established, and the three-dimensional grain selection bond was designed again by means of two-dimensional coupling optimization parameters.

## 1. Introduction

Single-crystal (SX) superalloys are widely applied to make industry gas turbines for its high temperature performance with a Fe–Ni or Fe–Co matrix. Thus, in order to improve the temperature bearing capacity and high temperature mechanical properties of turbine blades, Ni-based superalloys are considerably researched. Ni-based single-crystal (SX) superalloys such as Mar M247L, CMSX-6, and CMSX-4 are used to make the blades due to the excellent high temperature performance. In comparison with equiaxed (EQ) and directional solidified (DS), SX is choosing due to the better performance of the <001> orientation. To obtain a single-crystal structure, grain selectors are applied in practice.

The structure of a selector is mainly composed of two parts: The starting block and grain selector. The starting block controls the orientation and growth dynamics of the grain, and the selector favors the growth of individual grains and ends up with an SX structure. In current practice, a three-dimensional (3-D) spiral selector, called a pig-tail, is always employed in the production of SX components. The geometry of the selector plays a critical role on the grain selecting efficiency [1,2,3]. One of the key parameters of the selector is the wax wire diameter. Previous investigation indicates that the smaller wax wire diameter, the higher grain selection efficiency of the spiral selector. However, with the decrease of a wax wire diameter, the strength of the wax-based spiral selector decreases, at a certain point, the spiral selector cannot support the SX casting [4,5,6,7,8,9]. The manufacture of the model for injecting such a 3-D selector is also complex, and the operation process is complex. Due to the structure of the spiral selector, the 3-D demolding is difficult and a vertical metallographic observation of the whole dendrite growth in the selector is limited and not easy to directly describe the corresponding grain selection mechanism [1,2].

In this paper, we used the three-dimensional reduction method to analyze the view C and Z based on the spiral grain selection mapping in the 2D projection [1,2]. The method of dimensionality reduction has advantages as below:

1. For the high-dimensional data difficulty of analysis, and dimensionality reduction can simplify the process.
2. Due to the sample complexity of the high-dimensionality, this method can reduce the cost and analysis time of the experiment.
3. Dimensionality reduction can increase the readability of data and facilitate the discovery of meaningful data.

**Figure 1:**
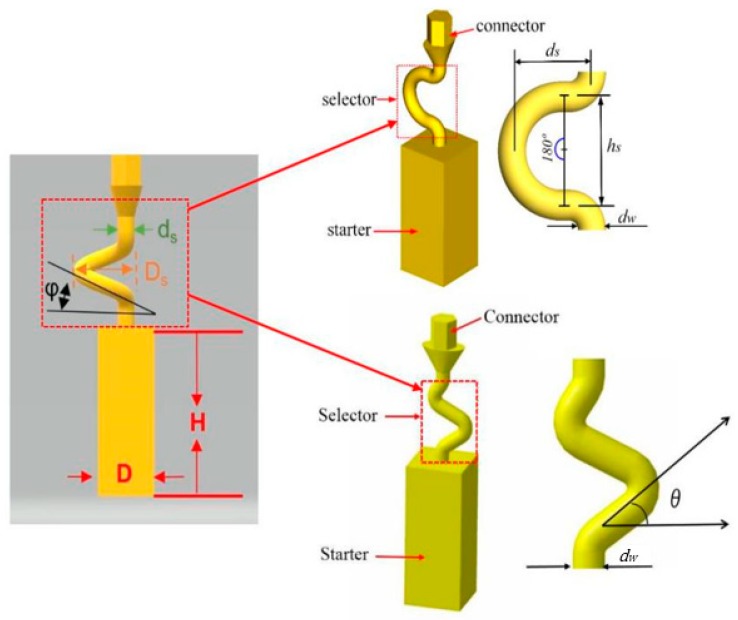
Illustration of the dimensional reduction method with a 2D selector. (d_w_: wire diameter, d_s_: eccentric distance, h_s_: pitch length, θ: take-off angle).

For the difficulties of research and analysis on a 3-D spiral grain selector and the possibilities to use a 2-D projection to simplify the 3-D structure, we have designed the C-form grain selector and Z-form grain selector with various geometry parameters (wire diameter, pitch length, and take-off angle) using a 3-D printing method to develop the optimized geometries of a 2-D grain selector. It is shown in Figure 1.

## 2. Experiments

### 2.1. 2D Grain Selector Design Produces

**Figure 2:**
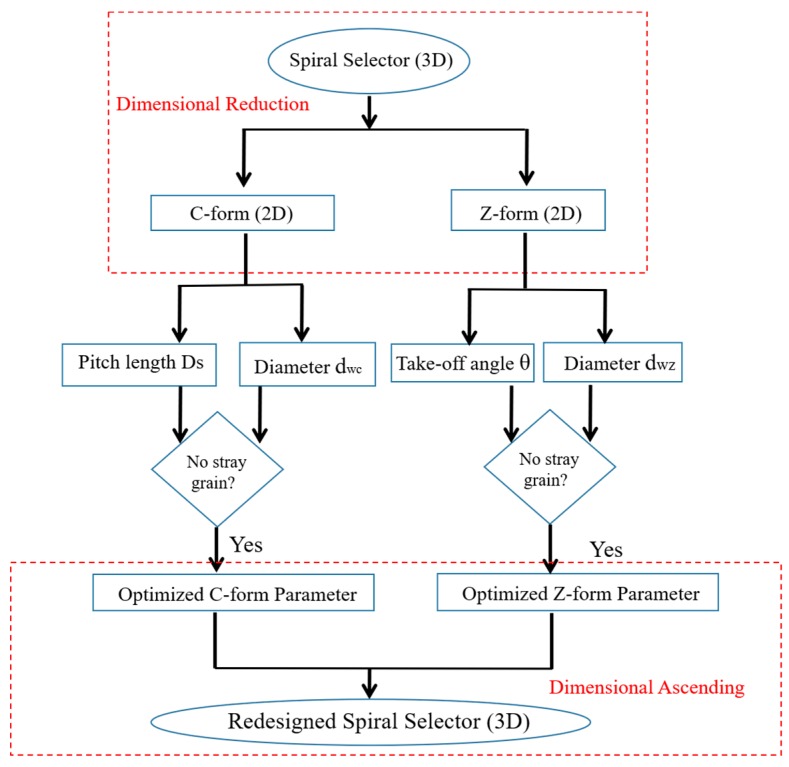
The flow chart of developing a high-efficient grain selector with the reduction dimensional method.

To construct the data of the three-dimensional spiral selector (pitch length, diameter, and take-off angle), each key parameter was investigated by a linear combination of its corresponding two-dimensional projection. The whole process was divided into four steps (as shown in Figure 2):

(1) Project a three-dimensional vision of a spiral selector into two-dimensional, resulting in a 2D view—C and Z;
(2) Experiment the C-form and Z-form grain selector to obtain the optimized parameters;
(3) Couple the optimized results of the two-dimensional model to project the corresponding three-dimensional spiral selector;
(4) Use the redesigned results in the spiral selector and employed in the single crystal blades casting to verify the experiment results.

### 2.2. Geometry Design of 2D Grain Selector: C-Form Grain Selector and Z-Form Grain Selector 

The 2D selector is composed of a starter block, a selector portion and a connector. In the research, the starter had a quadrangular shape with parameters of 10 mm (L) × 10 mm (W) × 30 mm (H). As it is shown in Figure 1.

#### 2.2.1. C-Form Grain Selector Design

The C-form selector part was designed with a fixing height (*hs*) and curved angle (θ = 180˚), but grains of different thicknesses chose routes (*dw* = 2.6–4.2 mm) and eccentric distances (*ds* = 6–20 mm) shown in Figure 1.

During the experiment, variations in two-dimensional geometric parameters were found to investigate the effect of different pitch length and diameters on grain selection. Table 1 summarizes the conclusion of the C type grain selector, a total of 16 cases were divided into two groups.

#### 2.2.2. Z-Form Grain Selector Design

The grain selector was designed with a varying take-off angle (θ = 15°–55°) and thickness (dw = 0.18–0.54 cm) shown in Figure 1.

2-D geometry parameters were varied to study the influence of a different diameter and take-off angle. A summary of the Z-form grain selectors used in this study is presented in Table 2, which includes 15 cases divided into two groups. The other 2 groups of grain selectors with different wire diameter and take-off angle are shown in Table 3 and Table 4 respectively.

### 2.3. Casting Experiment

Superalloy CM247LC was used in this study. Table 5 lists the chemical composition of CM247LC.

**Figure 3:**
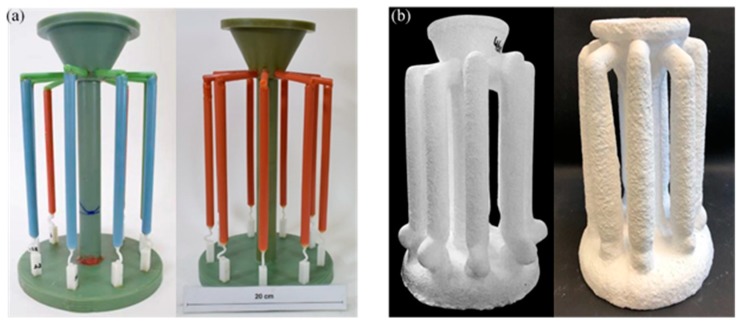
Images of the wax model (**a**) and the shell mold (**b**).

To study the influence of the shape of the selector on the grain selection, the wax bar composed of 2D grain selectors in different sizes and the cylindrical rod with 20 mm (D) × 150 mm (H) were injected as a whole. The parts were arranged around a center of gravity, forming a wax mold. Then, the wax group was immersed in a water-based ceramic mortar with different viscosities and painted with alumina mortar of different sizes. We repeated the whole processes until the shell mold wall thickness was up to 7–8 mm. After drying, the mold patterns were dewaxed. Thereafter, the mold was then sintered to remove the remaining wax and the shell strength was increased. The wax model and shell mold are shown in Figure 3. At last, the shell mold was mounted on a water-cooled copper cooler in a Bridgman furnace. In the process of investment casting, the shell mold was raised to a cylindrical heater, and heated to 1470 °C. After equalizing the furnace temperature, the melted alloy was heated to 1500 °C and poured into the mold. The mold was withdrawn from the furnace for the grain growth in a rate of 3 mm/min. As the temperature of the heater dropped below 300 °C, the vacuum was released, and then the mold was removed. Finally, we knocked out the mold and the units and cluster were separated. 

### 2.4. Microstructural Characterization

After removing the ceramic debris, the units were etched to reveal the macrostructure using a 50% H_2_O_2_ + 50% HCl etchant. The selectors were then cut longitudinally and transversely and etched using a 60 mL C_2_H_5_OH + 40 mL HCl + 2 g (CuCl_2_∙2H_2_O) etchant to show the dendrite structure in the selector. The structure of a dendrite is relatively complex, we mainly used the scanning electron microscope (Foundry Institute, RWTH Aachen University, Aachen, Germany) to study the evolution trend and dynamic change of dendrite structure.

## 3. Results and Discussion

### 3.1. Effect of C-Form Selector Parameters on Grain Selection

**Figure 4:**
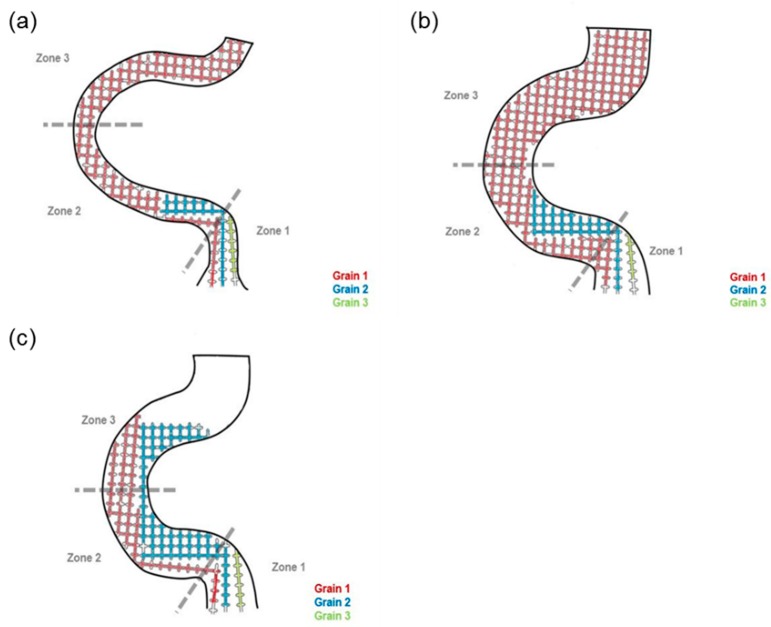
Grains (1, 2, and 3) growth and the selecting mechanism of the selector varying in diameter: (**a**) 2.6 mm, (**b**) 3 mm, and (**c**) 6 mm.

**Figure 5:**
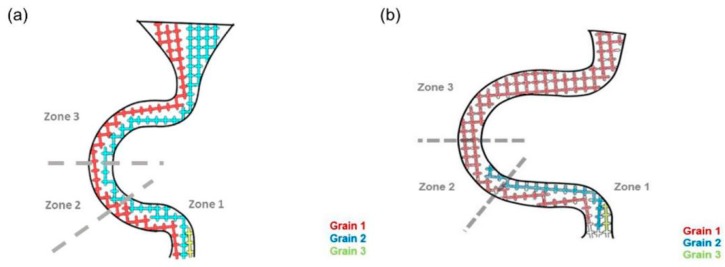
Grains (1, 2, 3) of geometrical blocking mechanism varying in pitch length: (**a**) 6 mm and (**b**) 8 mm.

In the C-form selector, the wire diameter (Figure 4) and pitch length (Figure 5) of the selector affected the grain selection.

Figure 4a illustrates the selection when the diameter was less than 3 mm. Grain 1 was growing at a faster rate than Grain 2 and Grain 3, so the further growth of Grain 2 and Grain 3 would be limited by the vertical dendrites of Grain 1. If so, Grain 2 and 3 would be stopped before reaching Zone 2, which means that increasing the wire diameter (less than 3 mm in diameter) had a negligible effect on the selection.

Figure 4b shows that when the diameter was 3 mm, Grain 2 had an opportunity to enter Zone 2 but hardly reached Zone 3 (the dendrite from Grain 1 could not block the dendrite from Grain 2 due to the larger selection diameter).

When the diameter increased (above 3 mm), shown in Figure 4c, the bound of the selection tunnel could not block Grain 2 from reaching Zone 3. Consequently, both Grain 1 and Grain 2 entered Zone 3. In that situation, Grain 2 would keep growing in the vertical direction until stopped by the dendrite of Grain 1 in Zone 3. When Grain 2 grew in the tunnel in Zone 3, stray grains occurred.

Considering the supporting strength of the selector, the wire thickness of 3 mm was the optimized result.

Figure 5a shows that when the pitch length is under 8 mm, the time needed for grains to reach Zone 2 was very short, so the effect of the tempo difference between Grain 1 and Grain 2 could not be obviously shown on such a short pitch length, so, Grain 2 would keep growing in the vertical direction until it is stopped by the dendrite of Grain 1 in Zone 3. Therefore, when the pitch length was shorter than 8 mm, the stray grain occurred.

Figure 5b shows that when the pitch length was above 8 mm (between 8 mm and 16 mm), Grain 2 had a chance to reach Zone 2, but due to longer pitch length and the beat difference between Grain 1 and Grain 2, it was almost impossible to reach Zone 3. 

**Figure 6:**
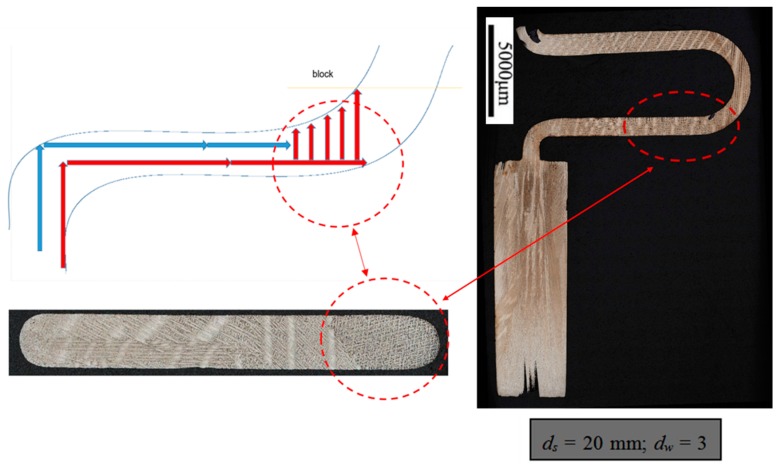
Schematic of the grain competitive and block mechanism with a long pitch length.

In order to illustrate the dendrite growth process with a long pitch length block mechanism, the growth model will be discussed in detail:

In the long pitch channel, due to the red dendrite close to the cool zone during the solidification, the red dendrite had a higher undercooling rate and it contributed to a higher ∆Tz, so the V(∆Tz) (red dendrite) was higher than the V(∆Tz) (blue dendrite). In the end, the red dendrite will block the blue dendrite as shown in Figure 6.

Considering the higher block efficiency long distance pitch length and the supporting strength of the grain selector, the 8 mm pitch length is recommended.

### 3.2. Effect of the Z-form Selector Parameters on Grain Selection

**Figure 7:**
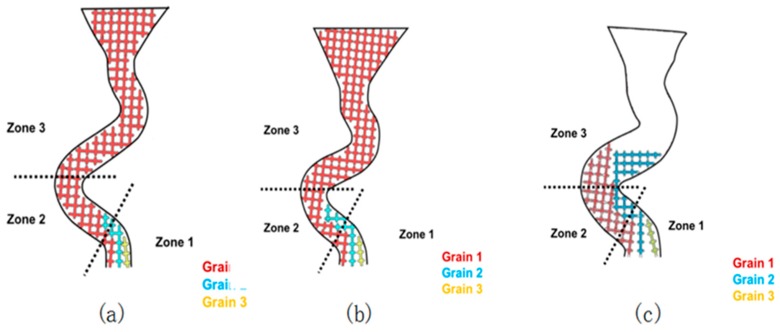
Grains (1, 2, and 3) growth and selecting in grain selectors varying in diameter: (**a**) 2.6 mm, (**b**) 3 mm, and (**c**) 6 mm.

**Figure 8:**
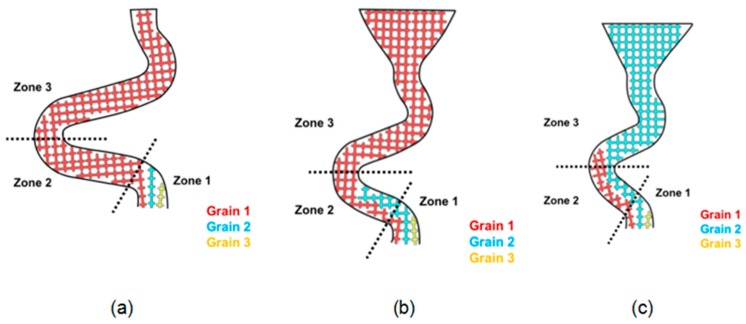
Grains (1, 2, and 3) growth and selecting in grain selectors varying in the take-off angle: (**a**) 15°, (**b**) 30°, and (**c**) 40°.

In the Z-form selector, the grain selection effect of the diameter (Figure 7) and take-off angle (Figure 8) are discussed as follows.

Figure 7a illustrates the selection when the diameter was less than 3 mm. Grain 1 grew at a faster rate than Grain 2 and Grain 3, so the further growth of Grain 2 and Grain 3 would be refrained by the vertical dendrites of Grain 1. In that case, Grain 2 and 3 would be stopped before reaching Zone 2, implying that increasing the wire diameter (less than 3 mm in diameter) decreases the selecting efficiency.

Figure 7b shows that when the diameter was 3 mm, Grain 2 had an opportunity to enter Zone 2 but hardly reached Zone 3 (the dendrite from Grain 1 could not block the dendrite from Grain 2 due to the larger selection diameter). 

When the diameter increased (above 3 mm), shown in Figure 7c, the bound of the selection tunnel could not block Grain 2 from reaching Zone 3. Consequently, both Grain 1 and Grain 2 entered Zone 3. In that situation, Grain 2 would keep growing in the vertical direction until stopped by the dendrite of Grain 1 in Zone 3. When Grain 2 grew in the tunnel in Zone 3, stray grains occurred. 

Comprehensively, a selector with the thickness of 3 mm was selected.

Figure 8a,b represent if the take-off angle was larger, Grain 2 achieved a higher position in Zone 2. When the take-off angle eventually reached 40°, as is shown in Figure 8c, Grain 2 had the possibility of reaching Zone 3 and finally grew through the grain selection tunnel. When the take-off angle exceeded 40°, a stray grain appeared.

Generally, the smaller the take-off angle, the higher the efficiency of the grain selection, but in practicality, the smaller take-off angle had weak rigidity in supporting the wax bar, so the take-off angle of 40° is recommended.

### 3.3. Effect of Wire Diameter Parameters on 2D Selector Applied in Spiral Selector

The experiment implies that the selecting efficiency could be enhanced by decreasing the thickness of the selector. Practically, the selector’s thickness of 3 mm in the C-form and Z form grain selector was applied and coupled 2D results to a 3D spiral model, the wire diameter 3 mm is recommended.

### 3.4. Effect of Take-Off Angle Parameters on 2D Selector Applied in Spiral Selector

The experimental results show that the smaller take-off angle had higher efficiency in grain selection. Considering the 2D grain selector stability, a thickness of 40° respectively of the selector take-off angle is recommended in spiral selectors.

### 3.5. Effect of Pitch Length Parameters on 2D Selector Applied in Spiral Selector

**Figure 9:**
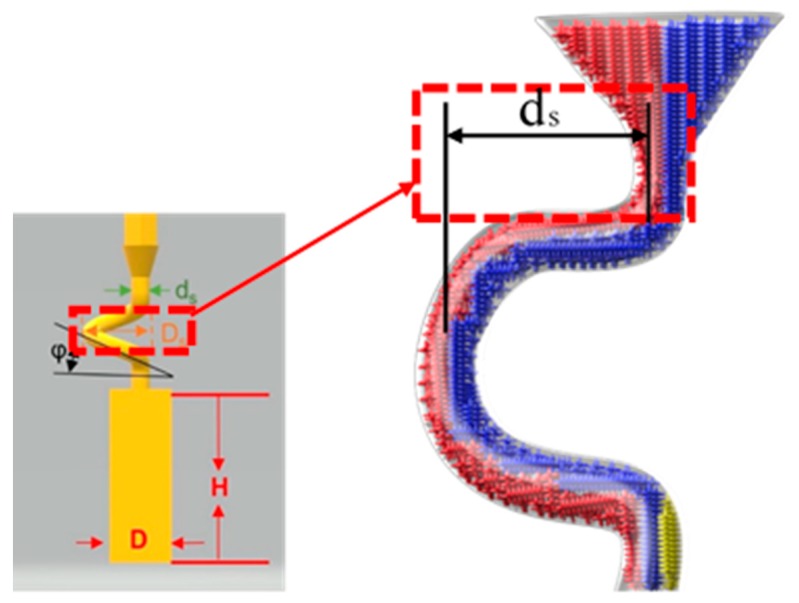
Schematic drawings of a 2D grain selector compared with a spiral selector in pitch length.

The experimental results show that the larger pitch length was more effective in two-dimensional grain selection. It is recommended to use 8 mm eccentricity respectively in practicality. As shown in Figure 9, the two-dimensional eccentricity distance was the two-dimensional linear projection of the three-dimensional spiral model. Therefore, the parameter of 8 mm should also be recommended in the three-dimensional spiral grain selector.

### 3.6. Combination of a 2D Parameter Using in 3D Grain Selection and Result Verification

As discussed formerly, three key parameters were optimized in the 2D model to describe the geometry of the spiral 3D model:

1. Wax wire diameter (ds) = 3 mm;
2. Pitch length (Ds) = 8 mm;
3. Take-off angle (θ) = 40°.

**Figure 10:**
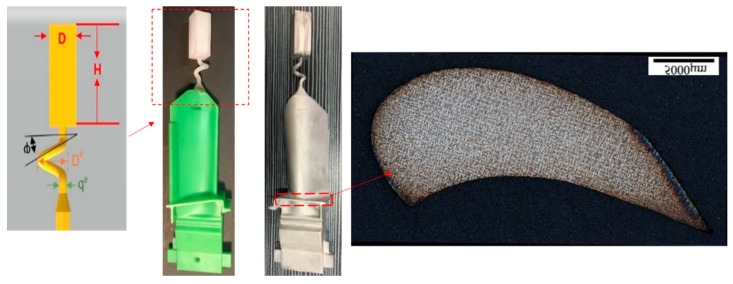
Flow-process diagram of the single-crystal (SX) turbine blade investment casting with a new spiral selector based on optimized 2D selector parameters.

Then the high-efficiency spiral selector was optimized by ascending the dimension shown in Figure 10.

## 4. Conclusions

An innovative 2D grain selector was studied experimentally using a three-dimensional reduction method. The results clearly showed for the selection behavior of the grain selection that a smaller take-off angle, a smaller thickness, and a larger pitch length were more efficient in grain selection. Considering the same geometry linear mechanism between the 2D and 3D model, the spiral selector’s take-off angle with 40°, a thickness of 3 mm, and pitch length of 8mm, respectively, are recommended. It was proposed that a redesigned spiral grain selector using the reduction method has higher-efficiency grain selection and was verified successfully in the turbine blade single-crystal casting. Furthermore, this method could provide a potential resolution and reference for future research.

## Figures and Tables

**Table 1:** Different thickness parameters of the C-form grain selectors: Group 1.

C-form grain selector with variant diameter	Single crystal		Stray grain					
	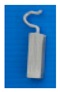	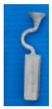	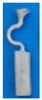	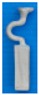	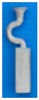	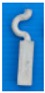	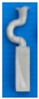	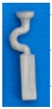
Stray Grain	No		Yes					
Probe	Cd1	Cd2	Cd3	Cd4	Cd5	Cd6	Cd7	Cd8
Diameter (cm)	0.26	0.30	0.34	0.38	0.42	0.54	0.62	0.66

**Table 2:** Different pitch length parameters of C-form grain selectors: Group 2.

C-form grain selector with variant pitch-length	Stray grain		Single crystal					
	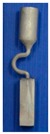	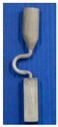	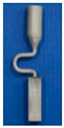	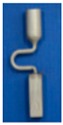	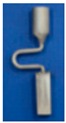	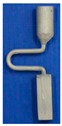	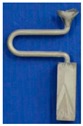	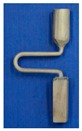
Stray Grain	Yes		No					
Probe	C1	C2	C3	C4	C5	C6	C7	C8
Pitch length	0.4	0.6	0.8	1.2	1.6	2.0	2.2	2.6

**Table 3:** Variation in the thickness of the grain selector portion: Group 1.

Z-form grain selector with variant diameter	**Single Crystal**				**Stray Grain**			
	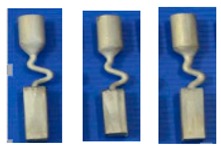				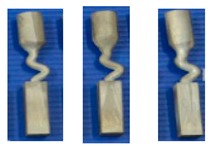			
Stray Grain	No				Yes			
Probe	1	2	3	4	5	6	7	8
Diameter (cm)	0.18	0.22	0.26	0.30	0.38	0.42	0.46	0.54

**Table 4:** Geometry of the selector design when varying the take-off angle: Group 2.

Z-form grain selector with a variant take-off angle	**Single Crystal**						**Stray Grain**		
	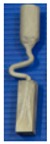	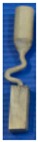	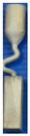	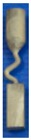	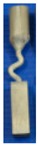	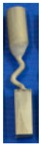	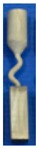	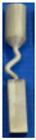	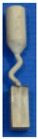
Stray Grain	No						Yes		
Sample	1	2	3	4	5	6	7	8	9
Take-off angle	15°	20°	25°	30°	35°	40°	45°	50°	55°

**Table 5:** The composition of superalloy CM247LC/wt.%.

Elements	Al	Ti	Cr	Mo	Co	W	Ta	Hf	C	Ni
wt.%	5.49	0.74	8.03	0.5	9.41	9.87	2.9	1.36	0.094	Bal.
